# Prevalence of Smear-Positive Tuberculosis among Patients Who Visited Saint Paul's Specialized Hospital in Addis Ababa, Ethiopia

**DOI:** 10.1155/2017/6325484

**Published:** 2017-08-21

**Authors:** Dinna Abera Nugussie, Getachew Ali Mohammed, Anteneh Tesfaye Tefera

**Affiliations:** ^1^Kotebe Metropolitan University, Addis Ababa, Ethiopia; ^2^Saint Paul's Hospital Millennium Medical College, Addis Ababa, Ethiopia; ^3^Medical Faculty, Addis Ababa University, Addis Ababa, Ethiopia; ^4^Institute of Biotechnology, Addis Ababa University, Addis Ababa, Ethiopia

## Abstract

**Background:**

Tuberculosis (TB) continues to be a health problem in both developed and developing countries, including Ethiopia.

**Objective:**

In this study, the prevalence of smear-positive tuberculosis among presumptive TB cases who visited the hospital was assessed.

**Method:**

Acid fast bacilli (AFB) test was performed on samples collected from 200 presumptive TB cases. Data were analyzed using appropriate statistical tools.

**Result:**

Among 200 presumptive TB cases, 10% (20 individuals) (60% were male and 40% were female) were found to be positive for the AFB. Of these AFB positive subjects, 11.2% and 6.3% were from urban and rural areas, respectively. Among 20 AFB positive cases, 45% (9), 45% (9), and 10% (2) were HIV positive, HIV negative, and with HIV status unknown, respectively. The highest AFB positive cases were found within age group between 25 and 44 years (70%) and followed by age above 40 years (30%). It was found out that 75% (15), 15% (3), 5% (1), and 5% (1) were unemployed, government employed, student, and nongovernment employed, accordingly.

**Conclusion:**

This study indicated higher level of AFB positive cases within age groups of 25–44 and 65–74 years and also exhibited higher prevalence of TB cases from urban areas.

## 1. Background

Tuberculosis (TB) is caused by* Mycobacterium tuberculosis*. It is a chronic bacterial contagious disease that continues to be major public health problem in the world. Most commonly, the disease attacks the lungs (pulmonary TB), and the lymph nodes, spine, or brain. Pulmonary TB is the only type of TB that can be passed on to others. It is transmitted from person to person via droplets from the throat and lungs of people with the active respiratory disease. If someone with TB coughs or sneezes, the bacteria in these tiny droplets can be inhaled into the lungs of another person, causing infection. Globally, in 2014 almost 9.6 million people fallen ill with TB; of this 5.4 million, 3.2 million and 1.0 million were men, women, and children, respectively. From these 9.6 million new TB cases, 12% of them were HIV positive. In the same year, it was estimated that 1.5 million people died of TB which included 1.1 million HIV negative and 0.4 million HIV positive cases [1].

Physicians differentiate between two kinds of TB infection: latent and active. In latent TB, the TB bacteria remain in the body in an inactive state. The bacteria do not cause symptoms and are not contagious, but it is possible that they can become active. In active TB, the bacteria cause symptoms and can be transmitted to others. It is generally estimated that one-third of the world's population is believed to have latent TB; and there is a chance that 10% of latent TB is becoming active TB [2]. This risk is much higher in people who have compromised immune systems, that is, people living with HIV or malnourished, or people who smoke [3]. Besides, TB affects all age groups and all parts of the world. However, the disease mostly affects young adults and people living in developing countries.

Ethiopia is one of the top sixteen countries in the world, and one of the top three in Africa, with regard to the number of tuberculosis (TB) patients. Over a third of the population has been exposed to TB. The annual risk of TB infection (ARTI) is at 2.2%. An estimated 377,030 Ethiopians (0.62% of the population) have active TB of all kinds, with more than 120,000 new cases in the 2003/04, nearly a third of which have smear-positive TB [4]. Recent study conducted by Elias et al. [5] estimated ARTI among rural community and was shown to be declined to 1.7%. The nation-wide survey study of TB prevalence (≥15 years) done by Kebede et al. [6] estimated the prevalence of smear-positive TB as 108/100,000 population. Another study carried out at Agaro Teaching Health Center, South West Ethiopia, indicated that the overall prevalence rate was found to be varying among male and female and on the basis of educational status of individuals [7]. On the other hand, TB associated with educational status and the number of smear-positive TB cases varied from level to level, which is 15/132 (11.4%), 7/94 (7.4%), 4/22 (18.2%), and 0/2 (0%) for uneducated, primary education, secondary education, and tertiary education, respectively [8]. So far, there is no published data to the authors' knowledge at Saint Paul's Specialized Hospital where there is association between the prevalence of TB and smear test. Therefore, this study was aimed at examining the prevalence of TB, among presumptive TB cases, its association with HIV, and the sociodemographic characteristics of the subjects.

## 2. Materials and Methods

### 2.1. Study Site and Design

The study was conducted between August and October, 2014, in Saint Paul's Hospital Millennium Medical College, which is found in Addis Ababa, the capital city of Ethiopia. Saint Paul's Hospital Millennium Medical College is one of General and Referral Hospitals in the country which all other kinds of patients with various illnesses including TB cases are referred to and treated at. The study was designed to investigate the incidence of the presumptive TB cases among those who visited the hospital with smear testing. An attempt was also done to relate the TB status of the subjects to variables such as sex, age, educational background, treatment history, and HIV status during the study period.

### 2.2. Study Population and Sampling Technique

Convenient sampling technique was implemented to include all suspected clinically identified presumptive TB cases who visited Saint Paul's Hospital during the study period. In this study, a minimum sample size of 185 presumptive TB cases was estimated by a single proportion formula using prevalence = 10.625% [3], 95% confidence interval, and 5% margin of error^10^. To compensate inadequate sample specimen and minimize errors due to sample collection, it was decided to consider an additional 8% of the minimum sample size which made the final sample size to be 200.

Individuals who were identified as presumptive TB cases were investigated for acid fast bacilli (AFB) from their sputum. A presumptive TB case was defined as any person who presented with symptoms or signs suggestive of TB, in particular cough of long duration (more than 2 weeks) [9].

### 2.3. Sample Preparation and Analysis

From every presumptive TB patient, three sputum specimens were collected and examined by microscopy using the Ziehl-Neelsen method of staining. Three sputum specimens (spot-morning-spot), 3 ml each, were collected from the study participants. The first sputum specimen was collected in the health facility (spot specimen) on the first day the patient visited the health facility. The second (morning specimen) was collected at home on the second day and delivered on the same day to the laboratory. The third sample was collected under the guidance of technician at health facility. The three specimens were processed in the laboratory for microscopic examination immediately.

From each of the three specimens, three direct smears were prepared, and then each of smear specimens was air dried, fixed, stained with Ziehl-Neelsen (Z-N), and examined under a microscope following standard procedure. After being examined under a microscope, the result was reported as positive for AFB or negative for AFB. Individuals with at least 2 positive smear results were diagnosed as sputum smear-positive and with active pulmonary TB cases. If any case was found negative, then it was excluded from the study.

Screening and confirmatory tests for HIV on blood samples taken from presumptive TB cases of those who visited Saint Paul's Hospital were done according to the national algorism for HIV testing indicated in Tarekegne et al. [10]. Briefly, blood samples taken from the presumptive TB cases were screened using HIV (1 +  2) antibody Colloidal Gold (KHB, Shanghai Kehua Bio-Engineering Co. Ltd., China), followed by HIV 1/2 STAT-PAK® (Chembio Diagnostics, USA) if positive. Where the result of STAT-PAK is discordant with KHB, Unigold™ HIV test kit (Unigold Rapid Test, Trinity Biotech, Ireland) [11] was used to determine the result.

### 2.4. Data Collection Instrument

Questionnaire was prepared to assess the prevalence in the study area. All participants who participated in the study were interviewed using a structured questionnaire. The questionnaire that included questions on sociodemographic characteristics, past family history of TB, and other risk factors was prepared. They were then clinically examined by the medical officer. For the kids who are involved in this study, their parents (mothers) were interviewed.

### 2.5. Data Analysis

The epidemiologic and laboratory data were collected, presented, and organized in the form of tables. Then, the data were analyzed using simple statistical tools like excel and percentage. AFB status and other major variables such as age, sex, treatment history, and HIV status were considered.

## 3. Results and Discussion

### 3.1. Results

In this study, the age group between 0 and 74 years were considered. The age of the study group was shown as 0–14 (6/200), 15–24 (18/200), 25–34 (61/200), 35–44 (58/200), 45–54 (223/200), 55–64 (11/200), and 65–74 (23/200) (Table 1). The prevalence of cough among participants for more than two weeks and one week was shown as 81% (162/200) and 19% (38/200), respectively. The prevalence of cough among males (52%, 105/200) was higher than that of females (24%, 48/200) (data not shown).

Our study indicated that the age group with the highest proportion of AFB positive was observed among persons aged 25–44 (70%) and 65–74 (25%) years (Table 1). In association to age group, our result also showed that age group within 15–24, 25–34, 35–44, 45–64, and 65–74 with 5%, 35%, 35%, 0%, and 25% was PTB positive, respectively (Table 1). The highest rate (70%) of PTB positive was observed among the age group of 25–44, and lowest rate of prevalence (0%) rate was observed among the age group of 45–64. In this study, the age group of 65 and above years was shown to be 25% (Table 1).

All presumptive patients provided three sputum samples for smear microscopy examination. From 200 subjects in this study, a total of 87% (174) volunteered to have HIV testing, of which 18.97% (33/174) were positive for HIV and 14.97% (26/174) were found with unknown status of HIV. Results related to HIV test among 20 AFB positive cases indicated that 45% (9), 45% (9), and 10% (2) were HIV positive, HIV negative, and of HIV status unknown, respectively. Of the 9 TB and HIV positive patients, 77.78% (7/9) were males and 22.22% (2/9) were females (Table 1).

From presumptive TB patients that visited the hospital, we found that 10% (20/200) were AFB positive. Of these AFB positive patients, 60% (12/20) were males and 40% (8/20) were females (Table 1). From 200 participating patients, 76% (152/200) were living in the urban areas and 24% (48/200) were from rural areas, of which 85% (17/20) and 15% (3/20) of the AFB positive patients were shown to be living in urban and rural areas, in that order (Table 2).

Our result showed that among 20 AFB positive participants 85% (17/20) had new smear-positive pulmonary TB and none of those were on anti-TB treatment before, whereas 15% (3/20) were already on anti-TB treatment. The study also indicated that, from a total of 20 AFB positive cases, 25% (5/20) had TB patients in their families (Table 2).

Results related to the educational status of AFB positive subjects showed that 10% (2/20) had no schooling, 40% (8/20) were elementary school (Grades 1–8) students, 35% (7/20) had secondary level (Grades 9–12) education, 5% (1/20) were a college level student, and 10% (2/20) were university graduates (Table 3).

Our result related to the employment status of the subjects with AFB positive smear indicated that 75% (15), 5% (1), 5% (1), and 15% (3) were unemployed, students, employed to nongovernmental organization, and employed to governmental institutions, respectively (Table 3).

### 3.2. Discussion

This study was a hospital based cross-sectional analysis which was conducted to assess the burden (prevalence) of TB among individuals visiting Saint Paul's Hospital during the study period. The study revealed that the prevalence of smear-positive TB among different gender (more in males), age groups (more in 25–44 and 65–74 years), educational background (more among lower level of education), family history (more among married), and employment status (more among unemployed). Different studies in Ethiopia have also indicated differences in prevalence of smear-positive TB among different gender and age groups [12–14], education [15], employment [16], and economic status [17]. Like other studies [18], in this study, the highest prevalence rate was observed in age group within 25–44 and 65–74, but the prevalence rate was very low in the age groups of 0–14 and 45–64.

The overall prevalence of sputum smear-positive TB in this study was indicated that 10% (20/200) among those presumptive TB patients visited the hospital. Another prevalence survey conducted by Gebre and Mimano [3] showed that 8.9% (33) from a total of 371 were confirmed as smear- or culture-positive PTB. Similar survey conducted in Jimma University indicated that the overall prevalence of TB among student population was 2.2% (49/2212) [19]. Even though family history is considered to be major risk factor for TB, in this study cases of AFB positive were found to be more among those patients without history of TB in their families (75%). However, in the study conducted by Kirenga et al. [20] among tuberculosis patients in Kampala, Uganda, family history (17.5%) was shown as one of the important TB prevalence risk factors.

The study done on juveniles in Karachi, Pakistan, indicated that TB prevalence rate was 2.2% (49/2212) for male and 3.9% (15/386) for female [21]. The observed high prevalence of TB (10%) in this study as compared to the three surveys may be due to combination of factors including overcrowding, poor health care facilities, and malnutrition. On the other hand, Banu et al. [22] in their study indicated that, from 466 inmates with suspected pulmonary TB and who tested with smear microscopy, 357 (77%) and 109 (23%) were found positive and negative for the test, respectively.

The prevalence of sputum smear-positive TB in this study was shown as 0.5% (1/200) and 3.5% (7/200) among age groups of 15–24 and 25–34 in that sequence. Unlike our study, another prevalence survey from a total of 6188 households reported rates of active TB as high as 8.5% and 6.5% among age groups of 15–24 and 25–34 years [18].

As it was documented in this study, high prevalence rate of TB cases was observed among males (60%) compared to females (40%). Similarly, the study conducted in India has shown clearly that the prevalence of pulmonary TB disease is significantly higher among males than females [23]. The same as this study, the high prevalence rate observed among male could be due to the fact that men are more exposed to the wider world as compared with women (especially in rural areas), with resultant greater social interactions with other people and greater risk of exposure to persons with TB disease, thus having a higher chance of becoming infected with TB [3, 20, 24]. The higher prevalence of tobacco smoking and alcohol consumption among males, as observed in studies conducted by different authors, could also be additional factors for the higher risk of TB disease observed among males [20, 25, 26].

Results of this study showed that urban residents (11.2%) have a higher prevalence of tuberculosis than rural residents (6.3%). Our result was found similar to the pattern shown in the study carried out in India [25]. This could be because urban residence reflects higher levels of population density and higher levels of out-door pollution in urban areas [27].

Our result also showed that there was higher prevalence of HIV among pulmonary TB patients 4.5% (9/200); and it was found out that the result was significantly different to the study carried out in China, Guangxi in which HIV prevalence among pulmonary TB patients was 0.5% (12/2,300) [28].

## 4. Conclusions

The overall finding of this study showed that high burden of TB among the urban population warrants appropriate measures to control TB. The higher prevalence of AFB positive sputum among males needs further exploration. Factors responsible for higher prevalence of TB should be investigated. Appropriate strategies for prevention (e.g., education and behavioral modification), targeted diagnosis and treatment are needed to strengthen TB control activities.

## Figures and Tables

**Table 1:** The prevalence of TB, HIV, and HIV-TB coinfection status with different age groups.

Age group	Total		AFB test				HIV						HIV & AFB			
			Positive		Negative		Positive		Negative		Unknown^†^		Positive		Negative	
	Number	%	Number	%	−Ve	%	Number	%	Number	%	Number	%	+Ve	%	−Ve	%
0–14	6	3.0	0	0	6	3.3	1	3.0	3	2.1	2	7.7	0	0	0	0
15–24	18	9.0	1	5	17	9.4	2	6.1	15	10.6	1	3.8	1^1^	11.1	0	0
25–34	61	30.5	7	35	54	30	9	27.3	41	29.07	11	42.3	2^1^	22.2	4	44.4
35–44	58	29	7	35	51	28.3	10	30.3	41	29.1	7	26.9	4^1^	44.4	2	22.2
45–54	23	11.5	0	0	23	12.8	5	15.2	16	11.3	2	7.6		0		0
55–64	11	5.5	0	0	11	6.1	1	3.0	8	5.7	2	7.6		0		0
65–74	23	11.5	5	25	18	10	5	15.2	17	12.1	1	3.8	2^2^	22.2	3	33.3

Total	200	100	20	100	180	100	33	100	141	100	26	100	9	100	9	100

^1^Male. ^2^Female. ^†^Those of whom retest was not done.

**Table 2:** Sociodemographic characteristics and nature of smear-positive patients who visited Saint Paul's Hospital, Addis Ababa, Ethiopia, from August to October 2014.

Variables	Number (%) of AFB positive	
	Number	%
Gender		
Male	12	60
Female	8	40
Marital status		
Single	2	10
Married	18	90
Location of living		
Urban^1^	17	85
Rural^2^	3	15
Nature of smear positivity		
New smear AFB positive	17	85
On anti-TB treatment	3	15
TB patient in the family		
Present	5	25%
Absent	15	75%

^1^Total subjects from urban residents were 152 out of 200 (76%) of which 11.2% were AFB positive. ^2^Total subjects from rural residents were 48 out of 200 (24%) of which 6.3% were AFB positive.

**Table 3:** Education status and type of employment of smear-positive patients.

Variable	Number	Percent (%)
*Level of Education*		
No school	2	10
Grades 1–4	5	25
Grades 5–8	3	15
Grades 9–12	7	35
College	1	5
University	2	10
*Type of employment*		
Student	1	5
Employed, nongovernmental	1	5
Unemployed	15	75
Employed, governmental	3	15

Total	20	100
